# Distinguishing the effect of lesion load from tract disconnection in the arcuate and uncinate fasciculi

**DOI:** 10.1016/j.neuroimage.2015.09.025

**Published:** 2016-01-15

**Authors:** Thomas M.H. Hope, Mohamed L. Seghier, Susan Prejawa, Alex P. Leff, Cathy J. Price

**Affiliations:** a Wellcome Trust Centre for Neuroimaging, Institute of Neurology, University College London, London, UK; b Institute of Cognitive Neuroscience, University College London, London, UK; c Department of Brain, Repair and Rehabilitation, Institute of Neurology, University College London, London, UK

**Keywords:** MRI, Stroke, Language, Outcomes, White matter

## Abstract

Brain imaging studies of functional outcomes after white matter damage have quantified the severity of white matter damage in different ways. Here we compared how the outcome of such studies depends on two different types of measurements: the proportion of the target tract that has been destroyed (‘lesion load’) and tract disconnection. We demonstrate that conclusions from analyses based on two examples of these measures diverge and that conclusions based solely on lesion load may be misleading.

First, we reproduce a recent lesion-load-only analysis which suggests that damage to the arcuate fasciculus, and not to the uncinate fasciculus, is significantly associated with deficits in fluency and naming skills. Next, we repeat the analysis after replacing the measures of lesion load with measures of tract disconnection for both tracts, and observe significant associations between both tracts and both language skills: i.e. the change increases the apparent relevance of the uncinate fasciculus to fluency and naming skills. Finally we show that, in this dataset, disconnection data explains significant variance in both language skills that is not accounted for by lesion load or volume, but lesion load data explains no unique variance in those skills, once disconnection and lesion volume are taken into account.

## Introduction

Recent brain imaging studies investigating the effect of white matter lesions on sensory motor and cognitive processing have described the severity of white matter damage in terms of ‘lesion load’, or the proportion of the target tract(s) that has been destroyed (Rorden et al., 2009, Zhu et al., 2010, Marchina et al., 2011, Fridriksson et al., 2013, Corbetta et al., 2015). Studies like this are increasingly complemented by those that measure disconnection explicitly (Thiebaut de Schotten et al., 2014, Kuceyeski et al., 2015). A tract whose volume is significantly compromised will also very likely be severed (or have many of its component fibres severed); in this case, lesion load is a reasonable proxy for disconnection in the fibre tract. But, it is also presumably possible for a tract to be completely severed by brain damage that spares most of that tract's volume—instances where disconnection is complete, but lesion load is still comparatively low. In cases like this, where the two measures diverge, analyses based on lesion load alone might actually mask a real association between damage to some tract, and the incidence or severity of some impairment of interest.

In what follows, we demonstrate that this divergence can and does happen in practice—and argue that the inferences made in at least one recent study might need to be refined as a result. Specifically, we (Analysis 1) reproduce a recent result associating fluency/naming skills in chronic stroke patients and lesion load in the arcuate fasciculus (Marchina et al., 2011), and (Analysis 2) illustrate how a tract disconnection analysis of the same data would drive a more positive inference than that made in (Marchina et al., 2011) about the potential role of the uncinate fasciculus in those skills. Finally (Analysis 3), we show that access to disconnection data significantly improves the quality of our models. This was quantified using hierarchical regression and a Bayesian measure, the Akaike Information Criterion.

## Method

### The PLORAS database

Our data are extracted from our *PLORAS* database which associates stroke patients, tested over a broad range of times post stroke, with demographic data, behavioural test scores from the Comprehensive Aphasia Test (CAT) (Swinburn et al., 2004), and high resolution T1-weighted MRI brain scans (Price et al., 2010). Stroke patients are only excluded if they are unable to consent for the study, have contraindications to MRI scanning or are unable to see or hear the stimuli required to assess their speech production abilities.

### Structural MRI data and lesion identification

Imaging data were collected using either a Siemens 1.5 T Sonata scanner, or a Siemens 3 T Trio scanner. In each case a T1-weighted 3D modified driven equilibrium Fourier transform sequence (Deichmann et al., 2004) was used to acquire 176 contiguous sagittal slices with an image matrix of 256 × 224 yielding a final resolution of 1 mm^3^: repetition time/echo time/inversion time = 12.24/3.56/530 ms and 7.92/2.48/910 ms at 1.5 T and 3 T respectively. From each patient's structural image, we created a binary lesion image, using a processing pipeline described in (Seghier et al., 2008).

### Language data

Each patient was assigned a behaviour score based on the tasks from the Comprehensive Aphasia Test. For ease of comparison across tasks, these raw scores are converted to T-scores, representing each patient's assessed skill on each task (e.g., describing a picture; reading non-words) relative to a reference population of 60 aphasic patients (Swinburn et al., 2004). The threshold for impairment is defined relative to a population of 27 neurologically normal controls such that performance below threshold would place the patient in the bottom 5% of the normal population. Lower scores indicate poorer performance. Taken in aggregate, the scores provide a reasonably detailed and complete characterisation of each patient's language skills.

Following the emphasis, in Marchina et al. (2011), on fluency and naming skills, our analysis focuses on T-scores assigned in the fluency and naming portions of the CAT. The fluency tests summarise scores on two sub-tests. In the first test, patients are asked to name as many animals as they can in one minute (i.e. a test of category fluency), and in the second test, patients are asked to say as many words as they can beginning with the letter ‘S’ in one minute (i.e. a test of letter fluency): scores are based on the number of correct utterances given. There are also two sub-tests in the naming assessment: in the first, patients must name each of 24 line drawings (pictures) of common objects, and in the second, they name 5 pictures depicting common actions (e.g. ‘eating’).

### Sample selection

Our sample selection procedure was designed to emulate the approach taken in (Marchina, Zhu et al. 2011). Patients were excluded if they: (a) were left handed or ambidextrous prior to their stroke; (b) were < 12 months post-stroke at assessment; (c) had evidence of other significant neurological conditions (e.g. dementia, multiple sclerosis); (e) did not speak English as a first language; (f) had suffered right or bilateral lesions, as assessed by a neurologist (APL), using the patients' raw T1-weighted scans; or (g) had suffered dispersed rather than focal damage. To make this last judgement, we excluded patients whose binary lesions occupied less than 100 contiguous voxels (2 mm × 2 mm × 2 mm)—reflecting the spatial scale at which the patients' scans are smoothed when compared to control data (Seghier et al., 2008).

**Fig. 1:**
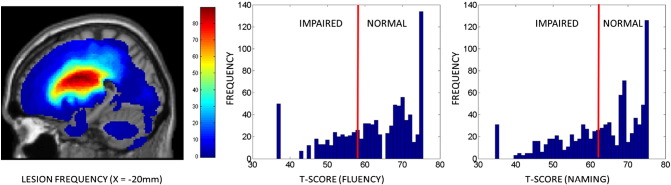
Lesion and language data. (Left) A sagittal slice of a lesion frequency image for the patients, at X = − 20 mm, and histograms of the patients' scores in the (middle) fluency and (right) naming assessments.

Following the approach taken in Marchina et al. (2011), we also excluded patients who, on the basis of their scores in the relevant CAT tasks, displayed either severe comprehension deficits or severe cognitive (i.e. non-linguistic) deficits. The CAT defines 5 assessments of comprehension (i.e. of spoken and written words and sentences, and also of written paragraphs), and 6 assessments of non-linguistic skills (line bisection, arithmetic, recognition and semantic memory, and gestural communication): we found the median score of all patients assessed as ‘impaired’ in each of these tasks, and excluded any whose scores fell below that median level in any task. A total of 142 patients met these criteria, 58 women and 84 men (age at onset: mean = 52.1 years, standard deviation = 13.2 years; time post-stroke: mean = 74.1 months; standard deviation = 80.0 months). Though all patients were judged to have suffered left hemisphere lesions, the volumes of those lesions were very variable (mean = 64.0 cm^3^, standard deviation = 72.2 cm^3^, minimum = 1.0 cm^3^, maximum = 387.7 cm^3^). Fig. 1 displays a sagittal slice of a lesion frequency image for the 142 patients (left), together with histograms of their naming scores in (middle) fluency and (right) naming assessments.

### White matter tracts

**Fig. 2:**
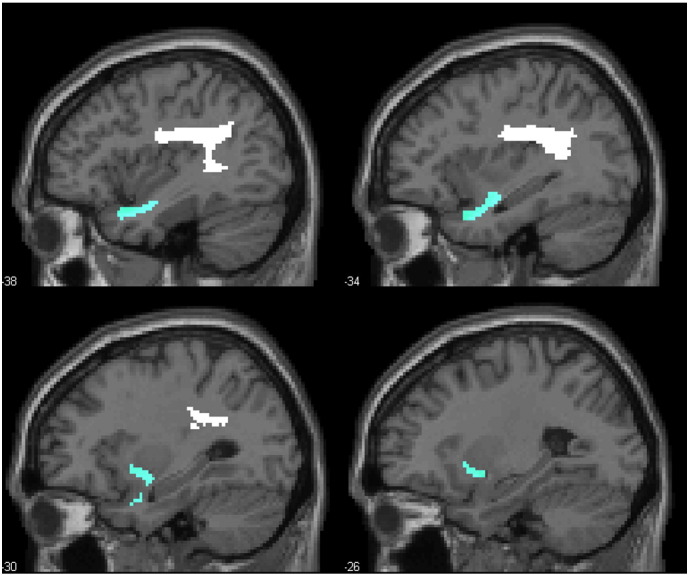
Sagittal slices of region masks for the arcuate and uncinate fasciculi. The arcuate fasciculus is displayed in white, and the uncinate fasciculus is displayed in blue.

Our analysis focused on two of the white matter tracts that were considered in (Marchina et al., 2011)—the arcuate and uncinate fasciculi. In that study, the authors constructed probabilistic images of the tracts (together with the extreme capsule, which we do not address here) from a separate data set of diffusion weighted images of 10 neurological normal controls. We extracted analogous images from the white matter tractography atlas published by Thiebaut de Schotten et al. (2011). In this atlas, white matter tracts were reconstructed from diffusion weighted images of 40 healthy subjects and defined in the same standard system of coordinates (MNI space) as the binary lesion images of our patients. We restricted our analysis to the volume where the tracts were observed in at least 25% of participants (see Fig. 2).

### Calculating lesion load and tract disconnection

Our analysis focuses on the distinction between lesion load in our images of the arcuate and uncinate fasciculi, and disconnection in those same two tracts, as both measures relate to stroke patients' scores in fluency and naming assessments. We define lesion load as the proportion of each tract image that is destroyed by, or overlaps with, a given stroke patient's binary lesion image: 0% if the tract is completely preserved by a lesion, rising to 100% when the tract is completely destroyed. Note that this is not the method employed in Marchina et al. (2011)—in that study, the authors sum the voxel intensities of the lesioned portions of each tract, to calculate a more probabilistic measure of lesion load. With our data, we found that the difference between these methods was largely immaterial, with both approaches yielding very strongly correlated results (r > 0.95 for both tracts). Since our tract disconnection measure depends on the use of binary tract images, we use the same images for our lesion load measure in what follows.

**Fig. 3:**
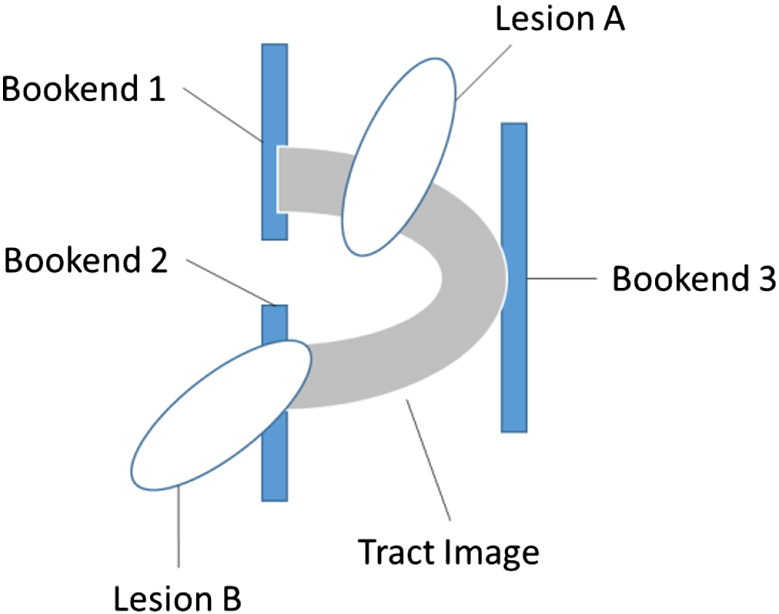
Schematic illustration of the measurement of disconnection. Both of the tract images that we consider are a single, connected object. Tracts are considered to be disconnected if a lesion either disconnects one part of the tract from another (Lesion A), or completely destroys one end of the tract (Lesion B). To make the latter measurement, we place a ‘bookend’ at each extreme of the tract, and search for instances where one bookend is separated from the others, after subtracting a given patient's lesion. Since both of the tracts that we consider here have two anterior projections from one posterior source, we add three bookends to each image before making our measurements. Disconnection occurs if, after subtracting a lesion, and of those three bookends are isolated from the others; in the figure, Lesion B disconnects bookend 2 from the others.

We used a bespoke algorithm to calculate whether each tract could be considered disconnected or severed by each patient's lesion. The core of the process draws on the ‘spm_bwlabel’ function, distributed as part of the Statistical Parametric Mapping software package (SPM8), which can be used to distinguish (and count) the ‘connected objects’ in a three-dimensional image. We use this function to characterise the effect of subtracting each patient's lesion image from each of our two tract images. Each of our tract images is a single, connected object: in the simplest case, where a lesion bisects a tract image in the middle but leaves its ends intact, the subtraction might raise the number of connected objects from ‘1’ to ‘2’ (see Fig. 3, Lesion A). To capture circumstances in which one or other extreme of a tract image is destroyed (see Fig. 3, Lesion B), we place a tract termination boundary (or ‘bookend’) at the extremes of each tract—three per tract, since both tract images have two anterior projections from one posterior extreme (see Fig. 2)—and search for instances where parts of those bookends are disconnected after subtracting a patient's lesion (see Fig. 3).

### Quantifying evidence in favour of lesion load versus disconnection models

We quantified relative evidence for the lesion load and disconnection measures using hierarchical regression and a Bayesian measure, the Akaike Information Criterion. In the hierarchical regression analysis, we use multivariate regression to measure the variance in both the fluency and the naming skills that is explained by: (a) a ‘lesion load model’ of the data, which includes lesion load in the arcuate and uncinate fascicles, and lesion volume; and (b) a ‘disconnection model’ of the data, which includes disconnection measures for the same two tracts, and lesion volume. Then we add: (c) the two disconnection measures to the lesion model; and (d) the two lesion load measures to the lesion load model, and use an F-test (implemented in the SPSS22 software package) to measure the significance of the R^2^ change in each case.

We use the Akaike Information Criterion (AIC) to quantify the relative evidence in favour of the lesion load versus tract disconnection models (i.e. those used in steps (a-b)), given our data, using the calculation implemented within the SPSS22 software package. The resulting AIC values (one for each model: AIC1 and AIC2) can be compared to generate a Bayes Factor (BF): BF = exp((AIC1 − AIC2) / 2). If the larger (worse) of the two AIC values is entered as AIC1, the result is a Bayes Factor which quantifies the relative likelihood that apparently better model (with the lower AIC value) really is the better model of the data. Following the popular convention proposed in Jeffreys (1961) to interpret these Bayes Factors, BF > 10 is interpreted as ‘strong’ evidence in favour of the chosen model.

## Results

### Lesion and language data

**Fig. 4:**
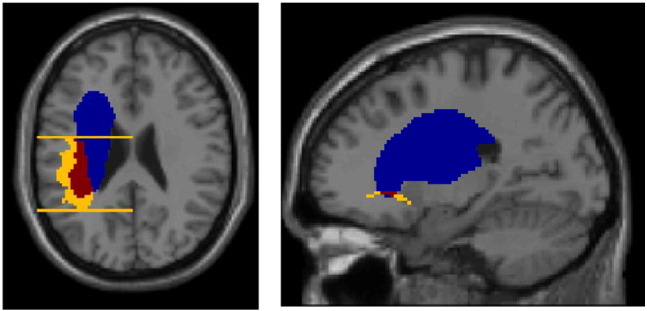
Two examples of divergence between lesion load and disconnection. (Left) High lesion load (45%) in the arcuate fasciculus but without disconnection; this figure includes two of three ‘bookends’ used to delineate the fascicle's most posterior and anterior extents. (Right) Disconnection of the uncinate fasciculus with only 5.5% lesion load. Colour code: blue = lesion; yellow = tract/tract ‘bookend’; red = intersection between lesion and tract.

Out of 142 patients, there were 76 and 53 patients whose lesions were judged to have severed the arcuate and uncinate fascicles respectively, and in both cases, the same patients' lesions also tended to destroy more of the tract images' volumes than those whose lesions caused no disconnection (arcuate: t = 12.7, df = 140, p < 0.001; uncinate: t = 12.4, df = 140, p < 0.001). Nevertheless, there were exceptions to this pattern, where the two measures diverged (see examples illustrated in Fig. 4): 27 patients had lesions that destroyed some part of the arcuate fasciculus (more than 20% in 6 cases), without disconnecting it, while 12 patients had lesions that destroyed less than 20% of the tract, but nevertheless left the tract disconnected (including two patients with less than 5% lesion load). There were 15 patients whose lesions destroyed some part of the uncinate fasciculus (the largest lesion load in this set was 18%) without disconnecting it, and 20 patients whose lesions destroyed less than 20% of the tract, but nevertheless left the tract disconnected (including another two with less than 5% lesion load).

#### Analysis 1: Replicating the association between lesion load in the arcuate fasciculus and fluency/naming

As in Marchina et al. (2011), we examined the relevance of lesion load in each of our two fascicles while controlling both for lesion load in the other fascicle, and for total lesion volume: we implemented the test with a 3-way analysis of variance (ANOVA), measuring just the main effects of each factor on fluency and naming skills respectively. In this analysis, lesion volume showed no significant association with either fluency (F = 1.94, p = 0.17) or naming scores (F = 1.79, p = 0.18). Similarly, there was no significant association between lesion load in the uncinate fasciculus and fluency (F = 1.90, p = 0.17), and only a non-significant trend for naming (F = 3.19, p = 0.08). But lesion load in the arcuate fasciculus did display significant main effects for both scores (fluency: F = 4.99, p = 0.027; naming: F = 8.31, p = 0.005). These results are consistent with those reported in Marchina et al. (2011).

#### Analysis 2: Replacing lesion load with tract disconnection information

Here, we repeated the previous analysis (i.e. a 3-way ANOVA) after replacing lesion load in each of the two tracts with a binary variable representing whether that tract was disconnected or severed by the patient's lesion. After making the replacement, we found that both tracts displayed independently significant associations with both language scores (fluency—arcuate: F = 8.77, p = 0.004; uncinate: F = 7.28, p = 0.008; naming—arcuate: F = 12.66, p < 0.001; uncinate: F = 5.23, p = 0.024). Lesion volume displayed a significant association with naming (F = 6.05, p = 0.015), but only a non-significant trend with fluency (F = 3.57, p = 0.061). Our disconnection analysis emphasises a potential role for the uncinate fasciculus in both fluency and naming skills, where the lesion load analysis implied no such role.

#### Analysis 3: Comparing lesion load and disconnection

In this analysis, we distinguish a ‘lesion load model’ from a ‘disconnection model’ of the fluency and naming data. The lesion load model includes two continuous predictors for lesion load in the arcuate and uncinate fascicles, whereas the disconnection model includes two binary predictors for disconnection of the same two fascicles. Both models also include lesion volume. We used SPSS 22 to calculate: (a) changes in the variance explained (R^2^) by each model, of the two language scores, when information from the other is added to it; and (b) the relative likelihood of each model, given those two sets of language scores.

Both models explain a significant proportion of the variance in both of the two language scores, though the lesion load model (fluency: R^2^ = 0.25, p < 0.001; naming: R^2^ = 0.31, p < 0.001) explains slightly less than the disconnection model (fluency: R^2^ = 0.29, p < 0.001; naming: R^2^ = 0.35, p < 0.001). Moreover, the variance explained improves significantly when disconnection data is added to the lesion load model (fluency: R^2^ change = 0.05, p = 0.007; naming: R^2^ = 0.04, p = 0.019), but not when lesion load data is added to the disconnection model (fluency: R^2^ change = 0.006, p = 0.56; naming: R^2^ change = 0.007, p = 0.47). In other words, the tract disconnection information appears to explain unique variance in these scores, whereas the lesion load information does not.

Using the Akaike Information Criterion (AIC), we also measured the relative likelihood of the lesion load and disconnection models, given the data. As expected given the linear regression results, the AIC values were lower (better) for the disconnection model than for the lesion load model for both language scores (AIC values for disconnection vs. lesion load: fluency: 529.47 vs. 536.13; naming: 503.83 vs. 508.75). Given these figures, the relative probability that the disconnection model is in fact the better choice can be calculated as: exp((536.13 − 529.47) / 2) = 27.9 for fluency (i.e. the disconnection model 27.9 times as probable as the lesion model, given the fluency data), and exp((508.75 − 503.83) / 2) = 11.7 for naming (i.e. the disconnection model 11.7 times as probable as the lesion model, given the naming data). Following the convention proposed by Jeffreys (1961), this is ‘strong’ evidence (i.e. ratio > 10) in favour of the tract disconnection model over the lesion load model for both language skills.

## Discussion

With a comparatively large sample of 142 patients with chronic left hemisphere stroke, we began by analysing the associations between fluency and naming scores with lesion load in (a) the arcuate fasciculus; and (b) the uncinate fasciculus. The associations between scores and lesion load reached significance in the arcuate fasciculus but not in the uncinate fasciculus, a finding that replicates the results previously reported in Marchina et al. (2011). However, when we consider whether each of the two tracts is disconnected, or severed, by each patient's lesion (Analysis 2), we found significant associations between both fascicles and both language scores. Moreover, at least in our data, there appear to be good reasons to prefer our disconnection-based characterisation to that based on lesion load. First, our tract disconnection information appeared to explain significant, unique variance in both language scores, but when tract disconnection and lesion volume were already taken into account, there was no apparent value in adding lesion load information to our model (Analysis 3). Consistent with that result, we found strong evidence to prefer the disconnection model over the lesion load model, for both the fluency and the naming scores.

Our results illustrate that inferences about the roles of white matter tracts depend on how tract damage is characterised: the disconnection-based characterisation was found to be better than that based on lesion load and there was no advantage of adding lesion load to the disconnection model. We do not claim that our particular measure of disconnection is the only or the best that might be used—indeed, it may very well be that stronger or more predictive signals could be garnered from more continuous measures of disconnection (e.g. (Kuceyeski et al., 2015)). Nor do we claim that every lesion-load-only analysis of white matter damage would be improved if our particular measure of disconnection were used instead. Lesion load has proved to be a powerful way to characterise white matter damage in recent studies, in the sense that it can drive strong associations with behavioural deficits (Wang et al., 2013), and even recovery from them (Wu et al., 2015). Nevertheless, our disconnection measure clearly adds something to the current analysis, over and above what was garnered from lesion load alone—something that may encourage a greater emphasis on measures of tract disconnection (however defined) in other similar studies.

Inferences about the roles of white matter tracts may also depend on other details of the analysis, such as the sample of patients and lesions being studied, and the ways in which other covariates are taken into account. In the current study, we included a relatively high number of participants, but we only considered the roles of each tract while controlling for lesion volume and lesion load or disconnection in the other tract. Whether characterised by lesion load or by disconnection, damage in any given tract may be strongly correlated with damage in other tracts or regions, not least those that border the tracts under study. In that context, our results (and others like them) probably cannot justify strong conclusions about the relevance of particular tracts unless much more is done to exclude or control for those covariates.

Another reason for caution stems from the age difference between our patient population, and the participants used to derive the tract images (18–22 years: (Thiebaut de Schotten et al., 2011)). This is in contrast to the study design of Marchina et al. (2011), where tract images were derived from age-matched controls. Though potentially significant, this difference appears to be largely irrelevant to the current study, because it does not prevent us from reproducing the key result from that older study (i.e. associating lesion load in the arcuate but not the uncinate fascicle with fluency and naming skills). Nevertheless, this difference encourages further caution when attempting to interpret the ‘real’ relevance of our target tracts to fluency and naming skills, based on the results reported here. In the current work, our aim has principally been to illustrate that two different ways of characterising white matter damage can drive different conclusions from the same data, and we have reproduced a recent lesion-load-only result (Marchina et al., 2011) to illustrate the practical consequences of that difference. But though our results are consistent with the claim, we cannot necessarily conclude that damage to our target tracts really has caused the fluency and naming deficits that our patients have suffered.

Measures of the presence or extent of brain damage—whether voxel-based or region-based—are a sensible starting point for most damage-deficit analyses, because they make few assumptions about the underlying mechanisms by which brain damage causes behavioural effects. As applied to white matter tracts however, the assumptions these methods do make may be misleading—at the region-level, because white matter disconnection can occur even if most of a tract's volume is preserved, and at the voxel-level, because no two patients' lesions need necessarily sever a tract at exactly the same place (e.g. Thiebaut de Schotten et al., 2014). Inferences based on measures of lesion load and measures of disconnection may tend to converge, as they have done in the current work as regards the role of the arcuate fasciculus. But our results with regard to the uncinate fasciculus illustrate that these measures can also diverge in practice, and that when they do, measures of disconnection can reveal damage-deficit associations that measures of lesion load may miss.
